# Working Women: Their Present and Their Future

**Published:** 1887-07-30

**Authors:** Walter Besant

**Affiliations:** Hon. Treasurer of the Committee of the Working-Women Conference. Adelphi Hotel, Adam Street, Strand, W.C.


### THE EDITOR'S LETTER-BOX.

[Our Correspondents are reminded that prolixity is a great bar to publication, and that brevity of style and conciseness of statement greatly facilitate early publication.]

### WORKING WOMEN: THEIR PRESENT AND THEIR FUTURE.

National Conference, Exhibition, and Demonstration.

Sir,—I beg to be allowed to make an appeal, through your columns, for assistance of a kind not usually asked; that is to say, of a kind which will certainly involve a considerable amount of personal activity and effort, and will call for the exercise of certain qualities of tact, sympathy, and discretion which are not always to be found.

It is proposed by certain persons interested in the present condition of working women, to hold a conference towards the end of the year, at which the facts of the female labour-market may be clearly and dispassionately set forth; the extent, area, and nature of the evils which undoubtedly exist, may be laid down with some precision; and, if that may also follow, remedies or alleviation may be found.

It is true that such a conference might have been held at once, for the mere purpose of discussing methods of relief for the well-known and widely spread diseases of over-work and under-pay. It is certain that there are here in London and other large centres thousands of women continually occupied in miserable toil for wretched wages, who never venture to hope for any cessation or relaxation of the life-sentence to which they were doomed at their birth. The conference might have proceeded on the assumption that these things have long been notorious. Yet the case for the women will be undoubtedly strengthened tenfold by the collection, the classification, and the publication of all the facts, apart from feeling, sentiment, hasty blame, and perhaps misplaced sympathy. This has never been done. True, figures and facts have from time to time been printed in certain Journals, and for certain trades, but the whole facts connected with women's industries—the hours and pay, the fluctuations of the demand, the competition of Germans and Jews, the effect of Free Trade, the freaks of fashion, the social and moral aspects of their work—have never been adequately investigated or treated. We do not aspire, of course, to collect and collate the entire facts in the brief time at command before the conference; but we propose to attempt an indication of them, as it were, on as wide a scale and in as thorough a manner as possible.

The committee formed by those who have originated this enterprise contains, among others:—The Hon. Miss Maude Stanley, Mrs. S. A. Barnett, Mrs. Rayner, Mrs. Heckford, Mrs. Alison, Mrs. Verney, the Rev. S. A. Barnett, the Rev. Brook Lambert, the Rev. Fleming Williams, the Rev. Hugh Price Hughes, the Earl of Meath, Mr. George R. Sims, Professor Stuart, Mr. Arnold White, Mr. Verney, Mr. A. A. Knight, and Mr. William Hill. We have divided ourselves for convenience into three sub-committees: one, on existing agencies, associations, trades unions, etc., established by or for working women; one, on the relations of work to the social life; and one, on wages, hours, and the condition of the work. We have drawn up a set of questions for each of these sub-committees. The questions are not intended to be put seriatim and verbatim, but are proposed as a guide to any who will assist us in this inquiry.

We invite, Sir, through your columns, the assistance of all persons who have the power of collecting information, and can get access to working women. There are in every parish, and belonging to every church and chapel, ladies who spend the greater part of their lives in and among the houses of the working-classes. It is more especially to these ladies, whose tact, disinterestedness, and sympathy have endeared them to the people, that we appeal for assistance. But the co-operation of men of all classes really interested in this movement is earnestly desired, and will be cordially accepted.

The committee have no views of their own to advance, and no theories to defend.—I remain, Sir, your obedient Servant,

Walter Besant,
Hon. Treasurer of the Committee of the Working-Women Conference.

Adelphi Hotel, Adam Street, Strand, W.C.

